# The Extracellular Matrix of Articular Cartilage Controls the Bioavailability of Pericellular Matrix-Bound Growth Factors to Drive Tissue Homeostasis and Repair

**DOI:** 10.3390/ijms23116003

**Published:** 2022-05-26

**Authors:** Tonia L. Vincent, Oliver McClurg, Linda Troeberg

**Affiliations:** 1 Centre for OA Pathogenesis Versus Arthritis, Kennedy Institute of Rheumatology, University of Oxford, Oxford OX3 7FY, UK; 2 Norwich Medical School, University of East Anglia, Norwich, Norwich NR4 7UQ, UK; o.mcclurg@uea.ac.uk (O.M.); l.troeberg@uea.ac.uk (L.T.)

**Keywords:** articular cartilage, pericellular matrix, mechanotransduction, osteoarthritis, growth factors, perlecan, heparan sulfate, extracellular matrix

## Abstract

The extracellular matrix (ECM) has long been regarded as a packing material; supporting cells within the tissue and providing tensile strength and protection from mechanical stress. There is little surprise when one considers the dynamic nature of many of the individual proteins that contribute to the ECM, that we are beginning to appreciate a more nuanced role for the ECM in tissue homeostasis and disease. Articular cartilage is adapted to be able to perceive and respond to mechanical load. Indeed, physiological loads are essential to maintain cartilage thickness in a healthy joint and excessive mechanical stress is associated with the breakdown of the matrix that is seen in osteoarthritis (OA). Although the trigger by which increased mechanical stress drives catabolic pathways remains unknown, one mechanism by which cartilage responds to increased compressive load is by the release of growth factors that are sequestered in the pericellular matrix. These are heparan sulfate-bound growth factors that appear to be largely chondroprotective and displaced by an aggrecan-dependent sodium flux. Emerging evidence suggests that the released growth factors act in a coordinated fashion to drive cartilage repair. Thus, we are beginning to appreciate that the ECM is the key mechano-sensor and mechano-effector in cartilage, responsible for directing subsequent cellular events of relevance to joint health and disease.

## 1. Articular Cartilage ECM

Articular cartilage lends itself well to investigating the function of the ECM. By volume, the ECM makes up around 90% of the tissue, with chondrocytes, the principal cell type in cartilage, contributing only 5–10% to tissue volume. Cartilage is devoid of blood vessels and nerves; thus, responses in the tissue are mediated by the chondrocytes alone, and presumably by mainly non-paracrine effects as the cells are separated geographically from one another with little cell-cell contact [1].

The ECM of cartilage is made up of two main components: type II collagen and the sulfated proteoglycan, aggrecan. In addition, there are many other less abundant collagens, proteoglycans, and glycoproteins that collectively constitute the matrisome [2,3].

**Figure 1:**
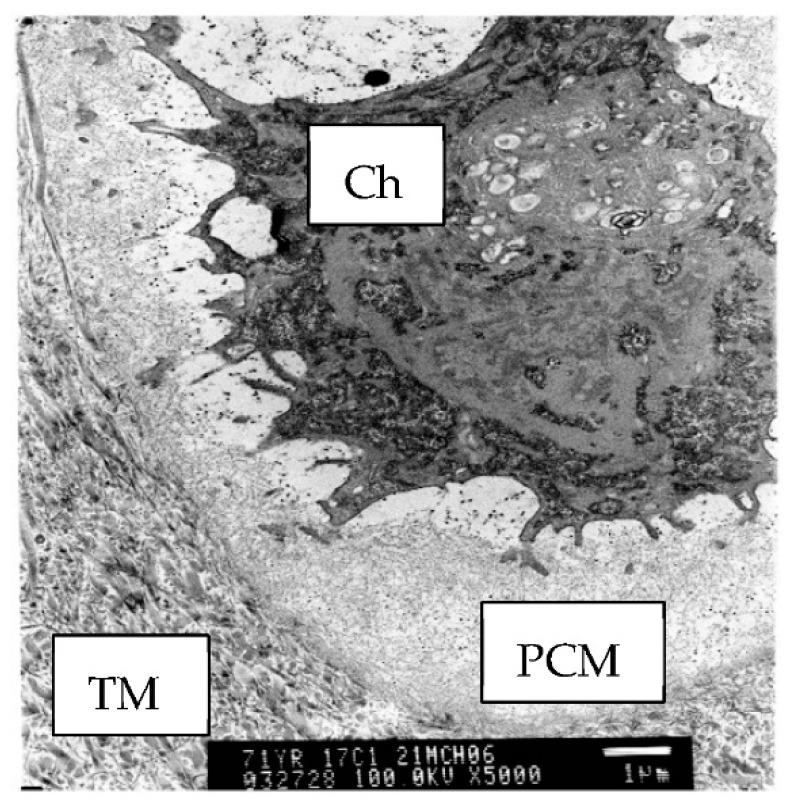
Electron micrograph of human articular cartilage showing a single chondrocyte (Ch) sitting within its pericellular matrix (PCM) and embedded within the type II collagen-rich territorial matrix (TM). Scale bar 1 μm.

**Figure 2:**
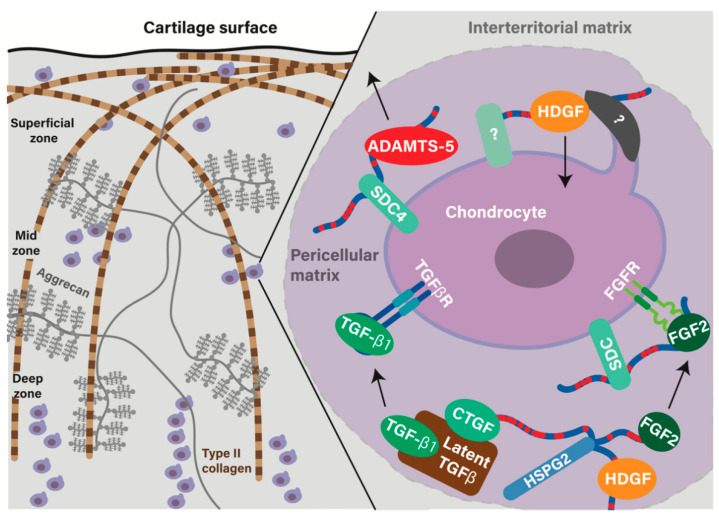
Schematic view of the cartilage extracellular matrix, highlighting the role of the pericellular matrix in chondrocyte signaling. Triple-helical type II collagen associates with type XI and type IX collagen (not shown) to form fibrils that extend through the territorial and interterritorial matrices of cartilage, ascending vertically from the deep zone, and becoming parallel with the cartilage in the superficial zone. Aggrecan, with its numerous negatively charged chondroitin sulfate GAG chains, draws sodium and water into the tissue. Together, these two macromolecules give cartilage its mechanical properties. Chondrocytes make up between 5–10% of the volume of cartilage. Immediately surrounding the chondrocyte is a pericellular matrix (right, purple shaded region), which is enriched in type VI collagen (not shown) and the heparan sulfate proteoglycan, perlecan. Perlecan sequesters a number of bioactive molecules including growth factors. Upon release, FGF2 binds to cell surface HS proteoglycans such as the syndecans (SDC) where it participates in the tertiary receptor complex. Connective tissue growth factor (CTGF) and hepatoma-derived growth factor (HDGF) are also bound to HS on perlecan. CTGF is covalently attached to latent-TGFß. Upon mechanical injury, the CTGF-bound latent complex is released, causing translocation to the cell surface where it binds to betaglycan (a cell surface HS proteoglycan) to activate TGFß and allow signaling. HDGF’s role in the joint is unclear. Receptors binding and internalizing HDGF in chondrocytes are unknown (labelled as question marks), but HS mediates HDGF internalization in other cell types. Key proteases have also been found to bind to HS: ADAMTS-5 is known to bind the HS side chains of syndecan-4, with shedding of the syndecan ectodomain proposed to promote ADAMTS-5 release.

Type II collagen is one of the fibrillar collagens, secreted as triple-helical homotrimers of Col2a1 that associate with type XI and IX collagens to form heterotypic fibrils within the tissue (Figure 1). The tertiary arrangement of these fibers is thought to contribute to their mechanoprotective role, lying parallel with the articular surface in the superficial cartilage and perpendicular to the surface in the deeper regions (Figure 2). The intermediate zone, just below the surface, is where the collagen is less well organized. This is the region that compresses preferentially upon loading of the tissue, effectively acting as the “crumple zone” of cartilage [4,5,6,7]. These different depth zones are also characterized by distinct protein composition [8].

**Figure 3:**
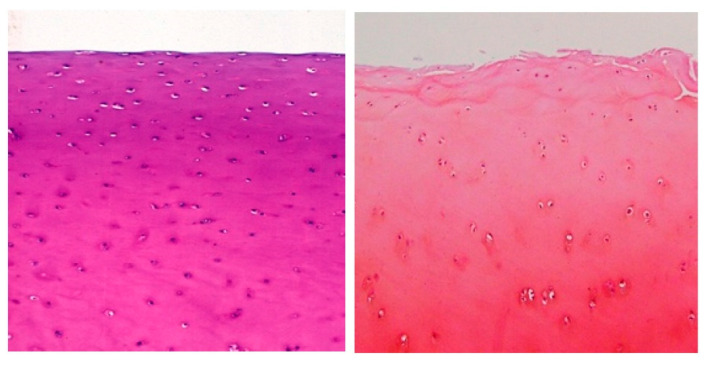
Normal (**left** panel) and early OA (**right** panel) human articular cartilage. Histological sections stained pink with safranin O (metachromatic dye). In normal tissue, chondrocytes are dispersed within the extensive extracellular matrix. Superficial cells, adjacent to the articular surface (top of image), are slightly flattened, reflecting the orientation of collagen fibers in this region. Early OA is associated with heterogeneity of proteoglycan staining (reduced near the articular surface with patchy increased staining deeper within the tissue). The articular surface loses its congruity and is associated with fibrillation and fissuring (20× magnification).

Aggrecan has a long core protein, along which are attached around 200 glycosaminoglycan (GAG) chains of mainly dermatan and chondroitin sulfate. These confer a high negative charge, pulling cations (principally sodium) and water into the tissue. Aggrecan is widely regarded as being a “sponge”, drawing in water and providing the tissue with the ability to compress and restore shape after the load is withdrawn. The fixed charge density that aggrecan creates forms the basis for many metachromatic dyes that are used in cartilage biology, e.g., safranin O and toluidine blue [9] (Figure 3).

## 2. The ECM in Osteoarthritis (OA)

In osteoarthritis (OA), degradation of type II collagen and aggrecan are regarded as key pathogenic processes. The role of metalloproteinases in disease was based originally on the observation that specific fragments of aggrecan were found within the synovial fluid of individuals with OA [10,11] and that cleaved type II collagen could be detected within the tissue using neo-epitope antibodies [11]. Identification of two major aggrecanases was made by purifying the enzyme responsible for the aggrecanolytic activity in IL1-stimulated bovine cartilage. This revealed a disintegrin and metalloproteinase with thrombospondin motif-4 (Adamts4) [12], with the second aggrecanase, Adamts5, subsequently identified by homology searching [13]. Using murine models of OA, Mmp-13 and Adamts5 were identified as key proteases in murine OA [14,15,16,17]. Genetic deletion of either leads to significant protection from joint damage in mice following surgical destabilization of the joint. Several pharmaceutical companies have developed aggrecanase inhibitors to attempt to treat OA (reviewed in [18,19]). Clinical trials in this area have largely failed, although the choice of primary clinical endpoints could be limiting the demonstration of efficacy in vivo. Activation of catabolic pathways in vivo after joint destabilization is highly mechanosensitive and appears to be related to abnormal shear stress at the articular surface, as mice that are able to put a compressive load through the joint but cannot flex the joint (as a result of sciatic neurectomy), do not upregulate metalloproteinases and do not develop OA [20,21].

Turnover of aggrecan and type II collagen are quite distinct. Aggrecan is rapidly turned over (like many proteoglycans). It is constitutively produced by chondrocytes and its regulation is highly mechanosensitive; it is down-regulated by physical inactivity (associated with cartilage atrophy) and increased upon mechanical load. It appears to be a primary driver of the mechanoadaptive responses in the tissue (reviewed in [22]). Type II collagen in cartilage was shown to have very low turnover in vivo in adult rats after injection of intra-peritoneal C^14^[glycine] [23,24]. Its striking stability was recently confirmed using the atomic bomb pulse method. These natural studies take advantage of a peak in atmospheric C^14^ as a result of atomic bomb testing in the 1950s. Only very stable proteins such as fibrillar collagens, synthesized at the time of high atmospheric C^14^ levels, are detectable in the tissue decades later [25]. This study revealed that C^14^ levels in type II collagen is taken from the articular cartilage at autopsy from healthy individuals were strongly associated with the stage of skeletal maturity during the 1950s. The result was almost identical in individuals whose joints were being removed at the time of arthroplasty surgery for OA, indicating that even when the cartilage is damaged, new type II collagen is not being replaced [25]. Low integration of type II collagen into post-natal articular cartilage is also demonstrated in mice using pulse SILAC labeling, in which stable isotope-labeled amino acids are delivered in the diet and their incorporation into tissues measured by proteomic analysis [3]. Using this technique, the incorporation of collagens and other matrisomal proteins was examined during healthy aging. It revealed a striking number of matrisomal proteins that are actively incorporated into articular cartilage and how these change (largely decrease) with age. Fibrillar collagens, in particular, were shown to be some of the least dynamic proteins of the adult tissue. Collectively, these results have led some to suggest that lack of renewal of type II collagen is the main reason for poor reparative activity in cartilage. It remains unclear whether joint off-loading treatments, such as joint distraction and high tibial osteotomy, in which there is apparent significant regrowth of cartilage, are mediated by type II, or another collagen [26,27,28].

## 3. Growth Factors of the Pericellular Matrix (PCM)

The ECM of cartilage is divided into three regions, the pericellular matrix (PCM), the territorial matrix (TM), and the interterritorial matrix (ITM) [29,30]. The ITM is the largest of the three zones and is rich in aggrecan and closely-packed collagen fibrils [29], providing the majority of tensile strength to cartilage. The TM is smaller in size (5–10 μm) compared with the ITM and contains less densely packed collagen fibrils that form a network protecting chondrocytes from the mechanical load into adulthood [31]. This region is often characterized by intense proteoglycan staining especially as OA progresses [32] or after injury [33]. The PCM is the thinnest of the matrix zones, forming a distinct 3–5 μm region surrounding each chondrocyte within the tissue. It has a distinct composition and is readily identified by electron microscopy by the absence of fibrillar collagens (Figure 1). The PCM of articular cartilage is rich in type VI collagen and the heparan sulfate proteoglycan perlecan. Although aggrecan must transit through the PCM to get to the TM and ITM, the PCM in mature cartilage appears to be relatively devoid of aggrecan [7]. Small leucine-rich proteoglycans such as decorin (Han 2019, Chery 2021) and biglycan (Chery 2021), and other proteins usually associated with basement membranes [34,35,36,37,38,39,40], are also present in the PCM. Decorin and biglycan may facilitate the interaction between type VI collagen of the PCM with type II collagen of the TM [35].

The PCM has quite distinct mechanical properties compared with the ITM and TM, and this lends itself to being an important mechano-regulatory hub in articular cartilage [41,42,43], a feature that is disrupted in OA [44]. Perlecan, type VI collagen, and decorin [45,46] contribute to the mechano-regulatory role of the PCM, and tissue homeostasis is severely compromised when they are disrupted [43,47,48] (and reviewed in [49]). One mechanism by which the PCM acts as a mechano-regulator in cartilage is by release, after mechanical injury, of a number of heparan sulfate-bound growth factors that are sequestered on perlecan [7,37,50,51] (Figure 2).

The best described of the PCM growth factors is fibroblast growth factor 2 (FGF2). This growth factor is known to have a high affinity for heparin and heparan sulfate [52], co-localizes in the PCM of cartilage with perlecan and can be released from the tissue with heparin degrading enzymes (which cleave the heparan sulfate chains) [36,37,53]. In mice, it is strongly chondroprotective as knockout mice develop accelerated spontaneous and surgically induced osteoarthritis [54], and delivery of FGF2 protects mice from OA [54,55]. Through further mouse knockout studies, chondroprotection appears to be mediated through FGF receptor 3 (FGFR3), rather than FGFR1 [56,57]. The FGFR1 neutralizing strategies in murine OA are able to suppress disease [58,59]. Interestingly FGFR3 and FGF18, a selective ligand for FGFR3, both come up as putative genome-wide association study targets in OA, such that low levels are associated with increased risk of disease [60]. This confirms that FGFR3 mediates important chondroprotective actions in humans as well as mice. This is further supported by recent phase II clinical trials in which a truncated form of recombinant FGF18 (sprifermin) was delivered intra-articularly [61,62]. At 3 years, sprifermin-treated individuals not only showed a delay in cartilage degradation but also an increase in cartilage thickness (measured by MRI) in OA affected and unaffected regions of the joint [62,63]. The intention to treat analysis did not show symptomatic benefit in the sprifermin group; however, a subsequent post hoc analysis, where only those individuals predicted to progress over time were analyzed, demonstrated symptomatic benefit [64]. Accepting the caveats associated with post hoc analyses, these results nonetheless indicate that harnessing this chondroprotective pathway represents the first disease-modifying approach in OA.

The PCM can be purified from cartilage by isolating the chondron (the chondrocyte within the PCM [34]) and then lysing the cell to release and remove intracellular contents. Using this method we performed a proteomic analysis of purified PCM from a normal human, osteoarthritic, and normal porcine tissue to identify other heparan sulfate-bound growth factors of the PCM. Four growth factors were identified in these analyses: FGF2, connective tissue growth factor (CTGF, also known as CCN2), hepatoma-derived growth factor (HDGF), and Cyr61 (also known as CCN1) [51]. CTGF turned out to be a novel latent TGFβ binding protein; sequestering latent TGFβ in the PCM until release, then transferring it to the cell surface heparan sulfate proteoglycan, betaglycan (also known as TGFβR3), which leads to its activation [51]. Although TGFβ is a strong regenerative and chondrogenic molecule, deletion of CTGF led to an increase in thickness of cartilage, hyperphosphorylation of Smad2/3, and protection against OA after surgical destabilization. This paradoxical result may be due to over-compensation by activin A, another TGFβ family ligand (unpublished results). The role and mechanism of action of HDGF and CCN1 are being explored. CCN1 has been associated with regenerative actions in other tissues [65]. In addition to the HS chains of perlecan, other molecules of the PCM have been shown in vitro to bind growth factors. For instance binding of FGF18 to the protein core of perlecan [66], Wnt3a to biglycan [67], and TGFβ to decorin [68]. Whether these factors are bound in this way in the native cartilage tissue and are similarly released upon tissue compression, is unknown.

When growth factors are released from the PCM under mechanical loading, they bind to the cell surface HS proteoglycans such as the syndecans and glypicans, enabling localization near or presentation to cell surface receptors and initiation of signaling. Little is known about the roles of glypicans in OA, but the expression of syndecan 1 and 4 have been shown to increase in OA cartilage [69,70,71], and syndecan 4 has been shown to promote ADAMTS-5 activity and cartilage damage [72].

## 4. The Role of Heparan Sulfate in Matrix Sequestration and Activation of Bioactive Molecules

Retention of growth factors and other bioactive molecules in the PCM can be altered by changes in the amount of sulfation of heparan sulfate proteoglycans. Changes in heparan sulfate structure have been observed in a number of tissues with aging, (e.g., in the heart and brain [73,74]) and with disease, (e.g., fibrosis [75]; cancer [76]). We found that heparan sulfate structure also changes in OA [77]; analysis of knee articular cartilage from age- and sex-matched healthy and OA patients showed that 45% of the 38 genes involved in heparan sulfate biosynthesis were aberrantly expressed in OA cartilage. This was accompanied by a change in heparan sulfate structure in OA cartilage, with increased sulfation on carbon 6 of glucosamine residues, which correlated with increased expression of *HS6ST1*, one of the three intracellular 6-O-sulfotransferases [77]. Otsuki and colleagues showed that elevated 6-O-sulfation is deleterious for the joint since deletion of either of the extracellular sulfatases SULF1 or SULF2 accelerated both spontaneous and surgically induced OA [78]. 6-O-sulfation likely regulates the activity of multiple proteins within the joint through a number of distinct mechanisms [79]. Increased 6-O-sulfation may increase the affinity of proteins for heparan sulfate both in the PCM and on cell surface proteoglycans such as syndecan, glypican, and betaglycan. This may either increase or decrease their biological activity at their high-affinity cell surface receptor, depending on whether binding to heparan sulfate augments downstream signaling (as has been shown for FGF2, where 6-O-sulfation is critical for ternary receptor complex formation [52,80]) or inhibits it (as has been shown for Wnts, where 6-O-sulfation inhibits receptor binding [81]). It will also affect how easily they are released from the PCM sequestered pool (see below for mechanism). Multiple growth factors and other bioactive proteins such as ADAMTSs and tissue inhibitors of metalloproteinases (TIMPs) are likely to be affected by increased 6-O-sulfation. Further research to identify the spectrum of heparan 6-O-sulfate-dependent proteins in cartilage is warranted. Agnostic approaches, such as those described by Thacker et al. for investigation of the heparan-3-O-sulfatome in neuronal cultures [82], may be particularly useful.

N-sulfation of heparan sulfate also appears to promote the development of OA. Severmann et al. found that mice heterozygous for N-deacetylase N-sulfotransferase 1 (NDST1), which adds sulfate groups on the glucosamine sugar, developed less severe surgically induced OA, with chondrocyte-specific inducible *Ndst1^-/-^* mice showing similar protection [83]. These protective effects were modest compared with those reported for *Sulf*-null mice [78], suggesting that changes in N-sulfation cause a less severe phenotype than changes in 6-O-sulfation. The importance of 6-O-sulfation is also illustrated by the fact that it is the only heparan sulfate modification that can be removed after synthesis, by the extracellular SULFs. SULF expression is also increased in OA chondrocytes, especially in chondrocyte clusters [84].

## 5. The ECM under Mechanical Load

The release of growth factors upon injury indicates that the matrix is not simply there to protect the chondrocytes from injury but to sense and respond to injurious load. For many years we puzzled over how this might occur especially as early studies had indicated that the release upon injury was not dependent on cell viability (dead cartilage also released growth factors upon injury) or temperature, suggesting that enzymatic activity was not involved. Recently we tested the hypothesis that changes in the concentration of free sodium were sufficient to displace growth factors from the PCM. This hypothesis was supported by three known properties of cartilage; firstly, the TM and ITM have an unusually high concentration of sodium (250–350 mM) [85] due to the high fixed charge density of aggrecan. Secondly, the interaction of the growth factor and heparan sulfate is an ionic interaction that can be overcome in vitro with sodium. Thirdly, the compression of cartilage is known to redistribute water from low to high stiffness areas thus changing the local sodium concentration.

We tested the hypothesis by showing that increasing the sodium concentration resulted in a lower threshold for PCM growth factor release and that depletion of aggrecan (by prolonged IL1 treatment) rendered the tissue unable to release growth factors upon injury. Upon compressive load, an increase in free sodium was visualized by ^Na^MRI in the region just below the articular surface. This region corresponded to a 300 um deep region of tissue that had reduced stiffness and reduced volume upon compression. This area was also the region where there was evidence of growth factor activation (Smad2/3 phosphorylation) upon loading [7]. Taken together our data support a very simple mechanism by which, without any energy expenditure, the articular cartilage is able to respond within seconds to injurious mechanical compression. It is possible that these pathways are activated in response to non-injurious loading but as these pathways are largely switched off in ex vivo tissue samples, we think that they are only activated when the load exceeds a given mechanical threshold for that individual. That threshold may vary from one individual to another and may change over time, i.e., mechanoadapt, according to activity levels and tissue stiffness.

Loss of aggrecan thus causes major problems for mechanically induced signaling in OA. Sodium can no longer be held within the TM and ITM and compression of the tissue are therefore unable to release growth factors bound in the PCM, despite the fact that they are abundant in OA tissue [7]. This effect may be compounded by increased 6-O-sulfation of heparan sulfate in OA, which would increase the binding affinity of the growth factor to the PCM and so further reduce growth factor liberation upon loading. Conversely increased Sulf1/2 in chondrocyte clusters in OA may enhance release in select regions of the matrix [84]. However, taken together there is likely to be a net loss of the ability to activate chondroprotective pathways and promote repair in damaged tissue. This data raises interesting questions about how restoring the charge in the tissue might recover the chondroprotective response. As the growth factors released from the PCM are likely to have different targets and responses, this approach is likely to be superior to adding just one growth factor back at a time. It would also ensure that growth factors were released only in the injured areas of cartilage rather than globally within the joint.

## 6. Other Mechanoresponses in Cartilage

The release of growth factors is not the only mechanism by which articular cartilage responds to injury. Numerous cell surface receptors such as integrins and ion channels have been implicated in mechanosensing activities in chondrocytes (reviewed in [86,87,88,89,90,91]). The primary cilium, thought to be a sensor of fluid flow in the renal tubule, has also been shown to modulate mechanical signals on the tissue in vivo and in vitro [92,93,94,95]. In addition, we have described a mechano-inflammatory response that appears to be selectively activated by shear stress at the articular surface [96,97]. This leads to the activation of inflammatory signaling and induction of inflammatory response genes in vitro and in vivo [20,98]. These pathways control matrix degradation through transcriptional and post-translational control of protease activity [99,100]. Although some of the pathways associated with this activation are understood, it is not mediated by a soluble factor (unpublished data) and we still do not understand how the signals are initiated upon shear stress—whether this is sensed by the matrix or by the chondrocyte itself.

## 7. Conclusions

We have come a long way from regarding the ECM as a scaffold for maintaining shape and protecting chondrocytes, to seeing it as the primary mechano-sensor and mechano-effector of cartilage. In essence, it is the ECM that directs the cell to respond in the face of a change in the mechanical environment. As the ECM ages, its ability to perform these mechano-adaptive and regenerative functions is likely to be blunted. For instance, the cross-linking of collagen is associated with ECM stiffness that impacts the mechanical responsiveness of the tissue [101], and reduced synthesis of individual matrisomal proteins occurs with healthy aging [7]. Both are likely to predispose to OA. Once OA develops these mechanisms are further impaired. As growth factor pathways are also proving to be successful therapeutic approaches in OA [62], these simple studies on the ECM appear to have revealed clinically relevant pathways that could have a significant impact on human disease. The important questions now are to understand whether and how the PCM growth factors and their matrix substrates work in an orchestrated fashion to promote the successful repair of articular cartilage.

## Data Availability

Not applicable.
